# Social norms don’t always work: An experiment to encourage more efficient fees collection for students

**DOI:** 10.1371/journal.pone.0177354

**Published:** 2017-05-24

**Authors:** Antonio Silva, Peter John

**Affiliations:** 1 Department of Anthropology, University College London, London, United Kingdom; 2 School of Public Policy, University College London, London, United Kingdom; Middlesex University, UNITED KINGDOM

## Abstract

The use of social norms has become the tool of choice for behaviourally informed interventions. However, it is still not clear for what type of contexts and populations is this intervention effective. This randomised controlled trial with 4298 students tests the applicability of social norms to improve the late payment of university tuition fees. We find that providing information to late payers does not increase their likelihood of paying. This finding highlights how the use of social norms may not always be an effective tool in influencing behaviour.

## Introduction

The use of descriptive social norms has increasingly become the tool of choice for behaviourally-inclined policy-makers and administrators. The idea is simple: inform people how many other people are doing an activity—and if the proportion is high—the remainder will be more likely to conform. The idea was initially tested by Cialdini and colleagues [1, 2] in a series of experiments involving litter and messages left in hotel rooms to encourage guests to recycle their towels [3]. The use of norms has been tested in a variety of contexts and behaviours, including encouraging people to settle fines and taxes, with a series of successful trials carried out by the UK tax authority [4], as well as trials on recycling and charitable giving [5, 6, 7].

One question that arises is the extent to which the use of descriptive social norms can be applied to a wider range of domains and populations. This study tests whether providing feedback on norms of university tuition fee payment encourages late-paying students to settle their fees. Students are often new to university so may not be aware of the norms of payment and, as a result, might underestimate the level of fee payment across the university. We hypothesised that late paying students are more likely to pay their fees if they are informed of the typical behaviour of other students; but we found that providing this information was not effective in improving the rate of payment of tuition fees.

In this paper, we briefly review the literature on social norms and the underlying theoretical framework, and set out the rationale and design of the study. We then report the results and discuss their implications for the research on social norms.

## Research on social norms

Humans are social beings who take behavioural cues from those around them. These cues are derived from social norms—common practices driven by shared beliefs about what is typical and appropriate behaviour. These informal understandings of rules emerge from cultural and social contexts and indicate how to behave in society; but they do not necessarily involve active reflection, as they are often unconsciously internalised [8, 9, 10].

There are two kinds of social norms, descriptive and injunctive. Descriptive norms are the perception of what people do and injunctive norms are what people approve or disapprove of [1]. Here we focus on descriptive norms with the assumptions that most people want to adhere to how the majority of people behave and tend to emulate the behaviour of people with perceived shared characteristics or where social distance is thought to be low [11].

In the case of private behaviours—like paying tuition fees—a large number of people act independently and don’t necessarily communicate their behaviour to others. This information asymmetry can then result in an individual being unaware of the normative behaviour and erroneously assume a different behaviour. The supply of the correct norm can then allow the individual to make a more informed choice. In this way, the supply of norms can be understood as a moral expectation that people aim to live to up [12], which can from a game-theoretic perspective facilitate the coordination of interactions [13].

Over the past decades, numerous studies have shown that the use of social norms can lead to behaviour change. One of the first applications of this approach was by Cialdini [1], who reduces littering using descriptive norms by varying the amounts of litter on the floor and using injunctive norms by placing handbills with different messages on cars’ windshields. In another famous study, messages left in hotel rooms in the U.S.A. indicating how many other people have recycled their hotel towels reduced the likelihood of guests asking for new towels [3].

There has also been an increasing use of social norms in public policy. Tax collection, in the UK and US in particular, has been at the forefront of using social norms to improve payment compliance. Messages using local norms of how many people pay tax significantly increase the numbers of late payers paying their tax [4, 14, 15]. Social norms have shown to be effective in several other contexts, including reducing prescription of antibiotics [16], curbside recycling [5], charitable giving [6], and energy consumption [17].

Despite most of the research in this area supporting the impact of social norms on behavioural change, there is some evidence that social norms are not always effective. In some cases, the provision of descriptive norms alone can highlight that a considerable number of people are, in fact, getting away with the non-normative behaviour, which reduces the effectiveness of the message [2]. Interventions based on average behaviours that inform people that they behave better than average can also result in a worsening of behaviour, as exemplified by energy conservation studies in which those with low levels of energy consumption increase their consumption after being informed that their average consumption is lower than their peers [18, 19, 20].

The effectiveness of social norms is also affected by the context of the interventions. For example, two studies of hotel towels, which were carried out in Europe [21, 22], failed to replicate the results of Goldstein et al [3]. As Bonher & Schlüter [23] highlight, these results may be due to different baseline behaviours: while norms may be effective in a North American context where the baseline is lower, in Europe where the baseline is higher the effectiveness of the intervention is attenuated.

In summary, while the majority of published social norm studies show a positive impact in shifting behaviour [24], a closer reading suggests they are not always effective and that context matters. As Dolan et al ([25], p. 2) argue, ‘*the impact of norms on behaviors such as charitable giving and productivity might be quite different to that of other behaviors such as resource use*. *The key difference in these behaviors is understanding the production function of the behavior*’. In other words, the findings of existing social norms studies do not show external validity to all venues and contexts, in spite of many published and unpublished studies showing positive results [24]. Thus, it is important to test the effectiveness of social norms interventions in a broader set of contexts. In this study, we test the hypothesis that providing feedback on the average payment rates of university tuition fees as part of a payment reminder will encourage late paying students to settle their fees to the university.

## Materials and methods

The study was designed and carried out at University College London (UCL), with the assistance of the Student Fees Office. The project was approved by UCL Research Ethics Committee (ID:3949/004). Consent was not obtained from the participants. Following the recommendation of UCL’s Research Ethics Committee, we sought permission from the student representative body, UCLU, to conduct the study.

The Fees Office sends out a reminder email to the students who have not paid their fees on time. The intervention kept the existing wording of the reminder email but inserted the sentence in bold “**OVER 90% OF UCL STUDENTS HAVE ALREADY PAID. PLEASE PAY THE AMOUNT DUE NOW.”** which appeared in a prominent place near the top of the email (see Appendix for the full text of the treatment and control emails). We ran a two-arm trial over two years with the Fees Office sending reminders in four rounds of emails in November 2013, February 2014, November 2014, and February 2015 to unique late paying students of each round (no subjects appeared twice in our data). The control was the normal email, but adjusted so that the only difference between the treatment and control was the insertion of the new text. The allocation of treatment was randomised and blocked by age and gender to ensure balanced samples. The original sample includes a total of 4374 emails sent, from which 76 emails were removed from individuals who were in debt twice over the two-year period of the study, and as result received two emails. We removed the 2^nd^ email from the final analyses, after checking that receiving the treatment email twice had no significant effect on the payment rates (β = 0.25 [-0.92;1.42], *p* = 0.67). The final sample included 4298 individuals who were sent 812 emails in November 2013, 1459 emails in February 2014, 652 emails in November 2014, and 1375 emails in February 2015.

Table 1 shows the characteristics of the sample, which is balanced across treatment and control for all the variables that we have available. We analysed the data controlling for rounds, year, gender, age, and the amount of initial debt. The experiment was powered to detect a treatment effect of 5% in line with other studies using social norms to increase payment rates.

**Table 1:** Descriptive statistics by control and treatment group on year, round (1^st^ round-November; 2^nd^ round-February), gender, age and initial amount of debt.

	Control	Treatment
	N	N
**Year 2013/14**	1135	1136
**Year 2014/15**	1000	1027
**November (1st Round)**	732	732
**February (2nd Round)**	1403	1431
**Male**	1046	1045
**Female**	1088	1118
	**Mean (S.D.)**	**Mean (S.D.)**
**Age**	27.1 (7.9)	27.0 (7.9)
**Initial Debt**	£3789 (£3244)	£3710 (£3232)
**Total**	**2135**	**2163**

We were not able to conduct a manipulation check to understand how the different messages affected the students. However, there was a surge in payments the following day after both the control and treatments emails were sent (27% of all payments occurred by the following day with the rate rapidly declining after the first few days), suggesting that students do read the emails and take action.

We use logistic regression models, with a binary outcome measure of payment by 14 days of reminder being sent, to estimate the effect of the intervention. The interpretation of the results is the same if OLS models are used (results from OLS models are available upon request to the authors). We first use a univariate model with only the treatment predictor and then use a multivariate model including covariates for gender, age, initial amount of debt, year (2013/2014 or 2014/2015), and round of reminders (November or February). The model specification is the following:

$$P_{i}=\alpha +\beta_{1}S_{i}+\beta_{4}X_{i}+u_{i}$$

Where P_i_ is a binary outcome measure set to 1 if a student pays the tuition fees debt 14 days after the reminder email is sent.; α is a constant; S_i_ is a binary treatment indicator set to 1 if student is randomly allocated to receive a social norm email, and 0 if receiving the control email; X_i_ is a vector of individual specific characteristics, including gender, age, debt amount, year, and month of the email (only present in model 2); and u_i_ is an error term. We present the marginal coefficients in Table 2, where β is the impact of being in the group receiving the social norm letter.

**Table 2:** Effect of intervention on payment of tuition fees. Model 1 shows logistic regression coefficients for treatment effect and Model 2 also includes covariates for gender, age, initial amount of debt, year (2013/2014 or 2014/2015), and round of reminders (November or February).

	(1) Univariate	(2) Multivariate
Treatment	-0.027 (0.061)	-0.034 (0.063)
Female (ref. Male)	-	0.121 (0.635)
Age	-	-0.035 *** (0.004)
Debt Amount	-	0.000 (0.000)
2014/15	-	0.105 (0.064)
February (ref. November)	-	0.904 *** (0.070)
Constant	0.274*** (0.044)	-1.053 *** (0.196)
Observations	4298	4296

Baseline probability: 0.43

## Results

**Fig 1:**
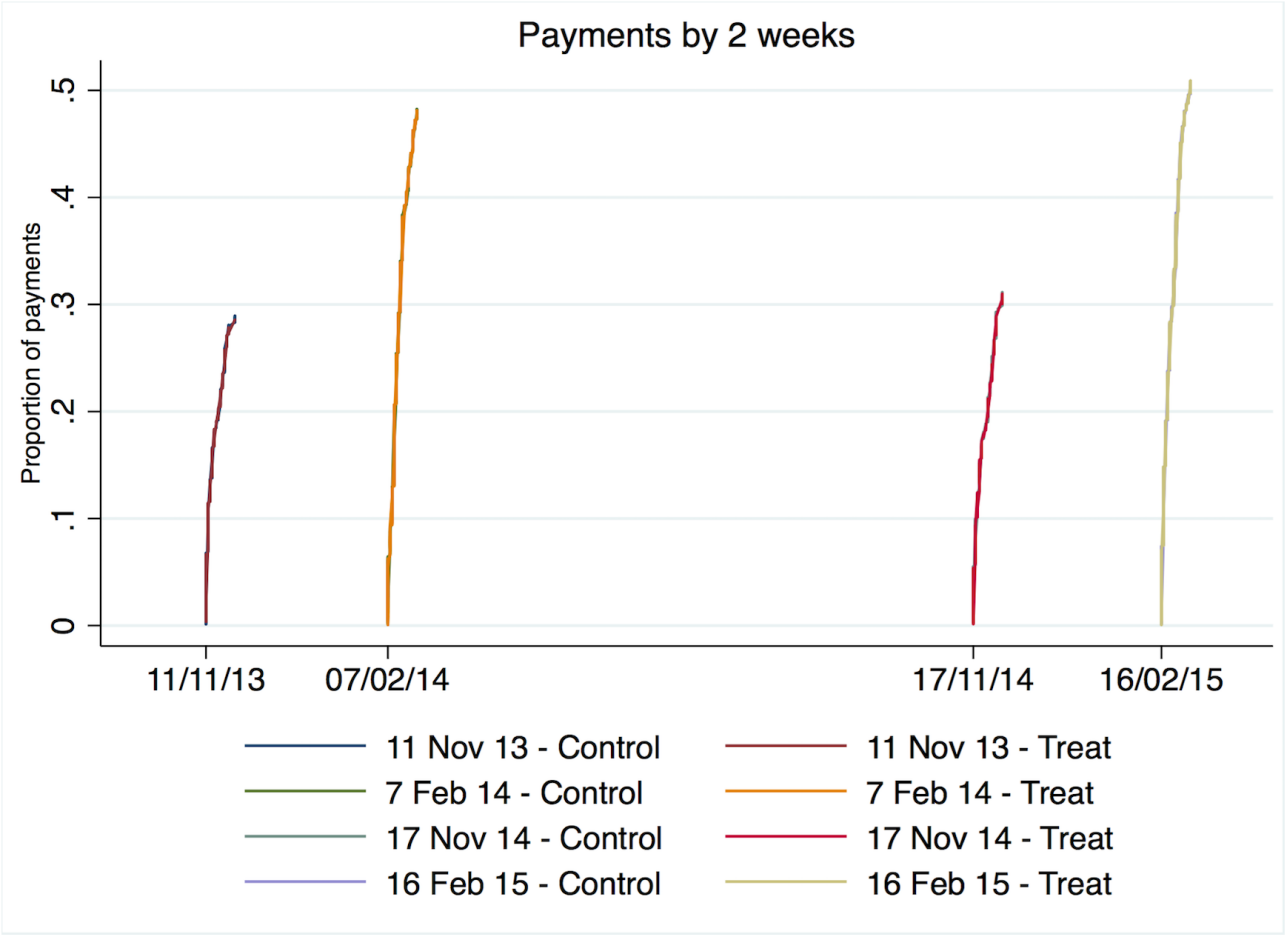
Payment rates over time. Decay in payment rates after email sent for control and treatment groups.

As Table 2 shows, we find no significant effect of receiving the modified social norm email on the payment of the tuition fees when compared to the normal control email. This non-significant result remains when including covariates in the analysis on gender, age, amount of debt, year, and round (Table 2). We also tested for the sub-group effects on gender, age, amount of initial debt, rounds, and year by running interactions of each factor with the treatment, but found no significant effect of treatment on any of the sub-groups. The payment rates over time also do not change between control and treatment emails (see Appendix, Fig 1).

We find that younger individuals are more likely to pay their late fees after receiving the reminder than older individuals. Individuals are also more likely to pay when receiving the 2^nd^ round emails in February compared to the 1^st^ round in November.

## Discussion

We find no impact of the use of social norms on the likelihood of payment of late university tuition fees. This study is a rare example of an intervention providing normative information that failed to change behaviour.

Below we propose a few potential explanations. Social norms are more likely to be effective in stable and homogeneous populations in which normative behaviour can provide a cue of what is the most efficient behaviour in a specific context. For example, providing the social norms of late tax payment at the local level were more effective in increasing the payment rate than providing the norm at the country level [4]. In contrast, the sample of our study—university students—is a heterogeneous and temporary population that may result in individuals not being influenced by normative information. In particular, students attending a London-based university come from a variety of countries and ethnic groups, especially at post-graduate level.

Whilst we find older students were less likely to pay on time, the intervention had no effect on any age group. However, the mean age of our sample is still lower than most of other social norm studies, which may indicate that the impact of this type of social interventions is less effective on younger people. The sample of late payers is also unusual as the majority of students get their fees paid automatically through their student loans, and as result are not part of our sample. The remaining may be late payers for several reasons, such as being international students who are not able to take out loans, students who decide not to take out loans, or students that simply don’t have the financial means to pay the fees. They may also be dependent on others to pay the fees whose behaviour they cannot control, such as parents, or a funding body that does not have a regular payment schedule. In summary, in addition to limited access to normative information, students are also likely to face budget and logistic constraints.

The effectiveness of social norms interventions depends on a population having a shared sense of what is the desirable form of behaviour. In heterogeneous populations, like the one in this study, this shared identity may not exist and anti-conformist bias may emerge, in which people prefer not to conform to the social norm as they don’t identify with the wider group [26, 27]. While we have not been able to define the reason behind the lack of impact of providing feedback on the normative behaviour in this context, our study highlights how social norms don’t always work, and the importance of determining the appropriate contexts where they may be effective.

## Appendix

### Treatment email

Student ID: 010207306–11/Nov/2013

Dear Student Name,

Your UCL Time Limited Enrolment has expired and our records show that you have not paid sufficient fees or provided the required sponsorship evidence to complete the enrolment process. The balance now due is £2,375.00.

**OVER 90% OF UCL STUDENTS HAVE ALREADY PAID**.

**PLEASE PAY THE AMOUNT DUE NOW**.

The easiest way to pay is on-line via the UCL website.

You can ask somebody to do this for you but they will need your Student Number (shown above). Alternatively, we accept payments by credit/debit card (sorry, no Amex, Electron or Diners Club), Bank transfer and UK Sterling Cheque. Full details of how and where to pay can be found at the Student Fees website

IF YOU FAIL TO ACT WITHIN THE NEXT TEN (10) WORKING DAYS WE WILL START TO APPLY SANCTIONS THAT WILL RESULT IN YOU BEING DENIED ACCESS TO SPECIFIC SERVICES & FACILITIES AND SUSPENSION OF YOUR REGISTRATION MAY THEN FOLLOW.

Students in debt to UCL are also to be prevented from attending a graduation ceremony, receiving official notification of results and being awarded their degree. If you have recently paid or provided sponsorship evidence, your Portico account will soon be updated. We advise that you check your Portico Account on a regular basis. If you forwarded evidence of external sponsorship (employer, government embassy etc) to UCL around the start of session that is not recorded on Portico, please email another copy to fees@ucl.ac.uk ensuring the email subject is "Evidence of Sponsorship". If you enrolled for the 2013/14 session on a Time Limited basis, this is the second email reminder that has been sent to your UCL account.

Please accept our apologies if you have received this email in error in which case you must contact Student Fees office (details below) as a matter of urgency to resolve this matter.

Any enquiries should be directed to the Student Fees Office by telephone or in person. The office is open for personal callers Monday to Friday from 10am to 4pm.

University College London,

Student Fees & Credit Control Section

Gower Street

London, WC1E 6BT

Tel: +44 (0)20 7679 4125 or 4128

### Control email

Student ID: 010207306–11/Nov/2013

Dear Student Name,

Your UCL Time Limited Enrolment has expired and our records show that you have not paid sufficient fees or provided the required sponsorship evidence to complete the enrolment process. The balance now due is £2,375.00.

**PLEASE PAY THE AMOUNT DUE NOW**.

The easiest way to pay is on-line via the UCL website.

You can ask somebody to do this for you but they will need your Student Number (shown above). Alternatively, we accept payments by credit/debit card (sorry, no Amex, Electron or Diners Club), Bank transfer and UK Sterling Cheque. Full details of how and where to pay can be found at the Student Fees website

IF YOU FAIL TO ACT WITHIN THE NEXT TEN (10) WORKING DAYS WE WILL START TO APPLY SANCTIONS THAT WILL RESULT IN YOU BEING DENIED ACCESS TO SPECIFIC SERVICES & FACILITIES AND SUSPENSION OF YOUR REGISTRATION MAY THEN FOLLOW.

Students in debt to UCL are also to be prevented from attending a graduation ceremony, receiving official notification of results and being awarded their degree. If you have recently paid or provided sponsorship evidence, your Portico account will soon be updated. We advise that you check your Portico Account on a regular basis. If you forwarded evidence of external sponsorship (employer, government embassy etc) to UCL around the start of session that is not recorded on Portico, please email another copy to fees@ucl.ac.uk ensuring the email subject is "Evidence of Sponsorship". If you enrolled for the 2013/14 session on a Time Limited basis, this is the second email reminder that has been sent to your UCL account.

Please accept our apologies if you have received this email in error in which case you must contact Student Fees office (details below) as a matter of urgency to resolve this matter.

Any enquiries should be directed to the Student Fees Office by telephone or in person. The office is open for personal callers Monday to Friday from 10am to 4pm.

University College London,

Student Fees & Credit Control Section

Gower Street

London, WC1E 6BT

Tel: +44 (0)20 7679 4125 or 4128
